# On the Strength of a 316L-Type Stainless Steel Subjected to Cold or Warm Rolling Followed by Annealing

**DOI:** 10.3390/ma13092116

**Published:** 2020-05-02

**Authors:** Marina Odnobokova, Zhanna Yanushkevich, Rustam Kaibyshev, Andrey Belyakov

**Affiliations:** 1 Institute for Physics of Advanced Materials, Ufa State Aviation Technical University, K. Marx 12, 450008 Ufa, Russia; odnobokova_marina@mail.ru; 2 Belgorod State University, Pobeda 85, 308015 Belgorod, Russia; yanushkevich@bsu.edu.ru (Z.Y.); rustam_kaibyshev@bsu.edu.ru (R.K.)

**Keywords:** austenitic stainless steel, cold/warm rolling, annealing, ultrafine grains, strengthening

## Abstract

The ultrafine-grained microstructures and their effect on the yield strength of a 316L-type austenitic stainless steel processed by large strain cold/warm rolling and subsequent annealing were studied. A kind of continuous recrystallization developed during annealing, resulting in the evolution of uniform ultrafine-grained microstructures with relatively high residual dislocation densities. The development of such microstructure at 973 K led to excellent combination of tensile properties including high yield strength (σ_0.2_ > 900 MPa) and satisfactory plasticity (δ > 15%). A unique power law function between the annealed grain size and the dislocation density with a dislocation density exponent of −0.5 was obtained for these continuously recrystallized microstructures. A physically justified explanation of the observed structural/substructural strengthening is introduced.

## 1. Introduction

Despite an excellent performance, austenitic stainless steels have a common disadvantage, which is associated with their relatively low yield strength inherent to a statically recrystallized microstructure after conventional processing [1]. The strength can be increased by structural/substructural strengthening. Reducing the grain size to nanometer range in the course of severe plastic deformation leads to a significant increase in the yield strength and the ultimate tensile strength [2,3]. Substantial grain refinement can be achieved through multiple cold working and fast annealing [4] that is assisted by austenite reversal [5]. Relatively low stacking fault energy in austenitic steels promotes grain refinement during large strain deformation [6]. On the other hand, such ultrafine-grained (UFG) and nanocrystalline materials have a serious drawback, that is, low ductility at room temperature. Moreover, austenitic stainless steels commonly experience strain-induced martensitic transformation during cold working [7] that alternates physical properties of the steels and may be detrimental for certain applications. Therefore, an appropriate heat treatment like multiple recrystallization annealing [8] or continuous recrystallization annealing [9] should be applied to obtain a suitable combination of mechanical properties of UFG steels. Promising strength-plasticity combinations have been observed in UFG steels after heat treatment resulting in UFG dual phase microstructure [10]. The evolution of UFG microstructure in cold worked austenitic steels upon annealing depends on the deformation mechanisms [11]. The development of UFG microstructure is commonly promoted by martensitic transformation [12] and repetitive type annealing [13]. The structural strengthening is commonly discussed in terms of Hall-Petch relationship [14,15]. However, the strength of UFG steels and alloys subjected to large strain deformation is rather difficult to express by a simple Hall-Petch equation due to the development of complicated hierarchical microstructure including well developed dislocation substructures with large internal stresses. There are several approaches to evaluate the strength after large strain deformation. Some of them consider the subgrain size as the main strengthening contributor [16,17,18]. Others include the grain boundary and dislocation strengthenings as independent and linearly additive contributors [19,20,21]. Despite certain achievements, however, the strength prediction for UFG metal materials processed by large strain deformation and subsequent annealing is still debatable. A unique inverse relationship between the flow stress and the grain/subgrain size that evolve in various UFG metals and alloys under conditions of severe plastic deformation [22] suggests the power law function between the grain size and dislocation density with a grain size exponent of −2 in such materials. This relationship between the microstructural parameters could be used for adequate prediction of strength properties in UFG materials processed by severe plastic deformation. However, the effect of post-deformation annealing on the relation among the microstructural parameters as well as microstructure-property relationship in UFG metals and alloys remain unclear. Therefore, using a 316L-type stainless steel processed by large strain cold/warm rolling and subsequent annealing, the present study is aimed at clarification of the relationship between the grain size and the dislocation density and at explanation of the effect of UFG microstructures on the strength.

## 2. Materials and Methods

A 316L-type austenitic steel (Fe-0.04% C-17.3% Cr-10.7% Ni-2% Mo-1.7% Mn-0.4% Si-0.05% S-0.04% P, all in wt.%) was investigated. This is one of the most widely used austenitic stainless steels for various applications from kitchen stuff to medical devices [1]. The steel samples with an initial grain size of 21 μm were subjected to plate rolling at ambient temperature (293 K) or at 573 K to total true strains of *ε* = 3. The rolled samples were annealed for 30 and 120 min at temperatures of 873 K to 1073 K followed by water quenching. Structural observations were performed on the sample sections normal to the transverse direction (TD), using a Nova Nanosem 450 scanning electron microscope (SEM) equipped with an electron back scattering diffraction (EBSD) analyzer incorporating an orientation imaging microscopy (OIM) system and a JEOL JEM-2100 transmission electron microscope (TEM, JEOL Ltd., Tokyo, Japan). The OIM images were subjected to clean up procedures setting a minimal confidence index of 0.1. The transverse grain size was evaluated on the OIM micrographs by a linear intercept method along the normal direction (ND), counting all boundaries with misorientations of θ ≥ 15°. The grain orientation spread (GOS) was mapped setting the grain tolerance angle of 5°. The dislocation density was measured by counting the number of intersections of individual dislocations in grain/subgrain interiors with TEM foil surface on typical TEM images observed under multiple beam conditions. The tensile tests were carried out on specimens with a gauge length of 12 mm and a cross section of 3.0 × 1.5 mm^2^ using an Instron 5882 testing machine.

## 3. Results

### 3.1. Microstructure Evolution

**Figure 1:**
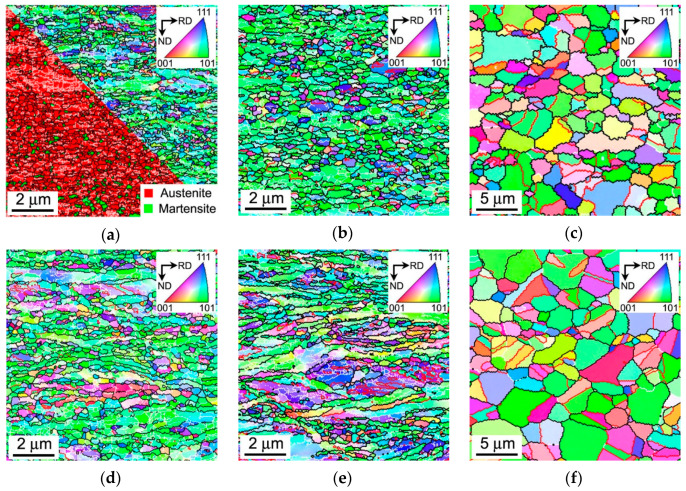
Typical microstructures in a 316L-type stainless steel subjected to cold (**a**–**c**) or warm (**d**–**f**) rolling and subsequent annealing for 30 min at 873 K (**a**,**d**), 973 K (**b**,**e**) and 1073 K (**c**,**f**). The black, white and red lines indicate the high-angle, low-angle and Σ3 CSL boundaries, respectively. The inverse pole figures are shown for the normal direction (ND).

The annealed microstructures that developed in the steel subjected to cold or warm rolling and subsequent annealing are shown in Figure 1. The main structural parameters are summarized in Table 1 along with same mechanical properties, which will be considered and discussed later. The microstructure evolution in the 316L steel during large strain cold/warm rolling has been detailed elsewhere [23]. Note here, the fraction of strain-induced martensite in the present cold rolled samples was 0.25 and the transverse austenite and martensite grain sizes were 80 nm and 70 nm, respectively, whereas UFG austenite with the transverse grain size of 160 nm was developed in the present warm rolled samples [23]. It is clearly seen in Figure 1a,d that annealing at a temperature of 873 K does not lead to significant changes in the deformation microstructures, suggesting that static recovery and partial austenite reversal (in the cold rolled sample, Figure 1a) are the main restoration processes at this temperature. Annealing at 973 K of the cold and warm rolled samples leads to the development of partial recrystallization occurring without grain boundary migration over a large distance (Figure 1b,e). The deformation grain/subgrain boundaries retain their wavy appearance caused by frequent micro-shear banding during the large strain cold/warm rolling. The grains with an average size below 0.5 μm retain elongated shape and contain large internal distortions as suggested by a gradual color change in the OIM micrographs. Recrystallization followed by grain growth at 1073 K leads to the formation of equiaxed grains with a size above 1 μm. The grain coarsening is accompanied by the development of annealing twins, which appear as special boundaries with dense coincident site lattices (i.e., Σ3 CSL boundaries indicated by red lines in Figure 1c,f) and a decrease in the internal distortions. In contrast to the samples annealed at 873–973 K, which retain their rather strong rolling texture, for example, < 110 >//ND, the samples annealed at 1073 K are characterized by almost random orientation (s. color variation in Figure 1c,f).

**Figure 2:**
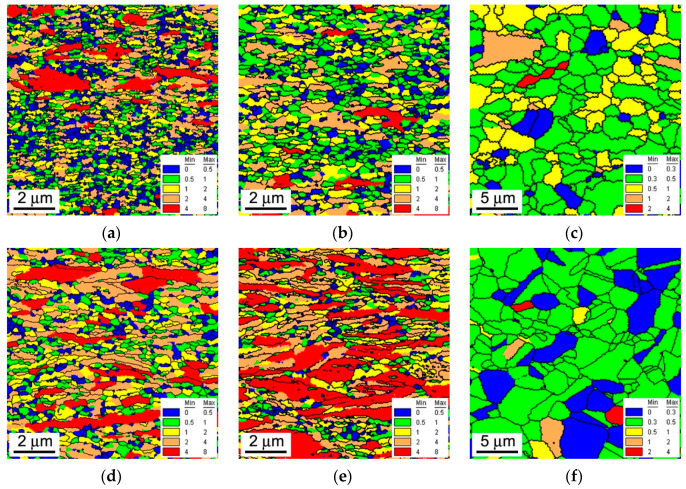
The grain orientation spread (indicated in degrees) in a 316L-type stainless steel subjected to cold rolling (**a**–**c**) or warm rolling (**d**–**f**) and subsequent annealing for 30 min at 873 K (**a**,**d**), 973 K (**b**,**e**) and 1073 K (**c**,**f**). The high-angle boundaries are indicated by black lines.

The corresponding OIM images showing the grain orientation spread in the annealed samples are presented in Figure 2. The samples annealed at 873-973 K are characterized by large grain orientation spread (GOS), which exceeds 5 degrees in many grains (Figure 2a–e). It should be noted in these figures that relatively large GOS values are observed even in the fine recrystallized grains with more or less equiaxed shape. The presence of such rather large GOS in the recrystallized grains is indicative of the continuous mechanisms of recrystallization. This is coincident with the current opinion that post-dynamic recrystallization in severely strained metallic materials with UFG microstructure always develops in continuous manner [24]. The development of continuous post-dynamic recrystallization does not completely release internal distortions in the recrystallized grains. Instead, the deformation substructures with relatively high dislocation density caused by cold/warm working are occasionally distributed in continuously recrystallized grains. A decrease in the rolling temperature promotes the post-dynamic recrystallization. Therefore, the present cold rolled and then annealed at 873–973 K samples in Figure 2a,b exhibit smaller GOS as compared to warm rolled samples in Figure 2d,e. An increase in annealing temperature to 1073 K encourages recrystallization resulting in remarkable grain coarsening. However, rather large GOS comprising 2 degrees in some randomly located grains suggests relatively high dislocation densities in the samples even after annealing at such a high temperature as 1073 K (Figure 2c,f).

**Figure 3:**
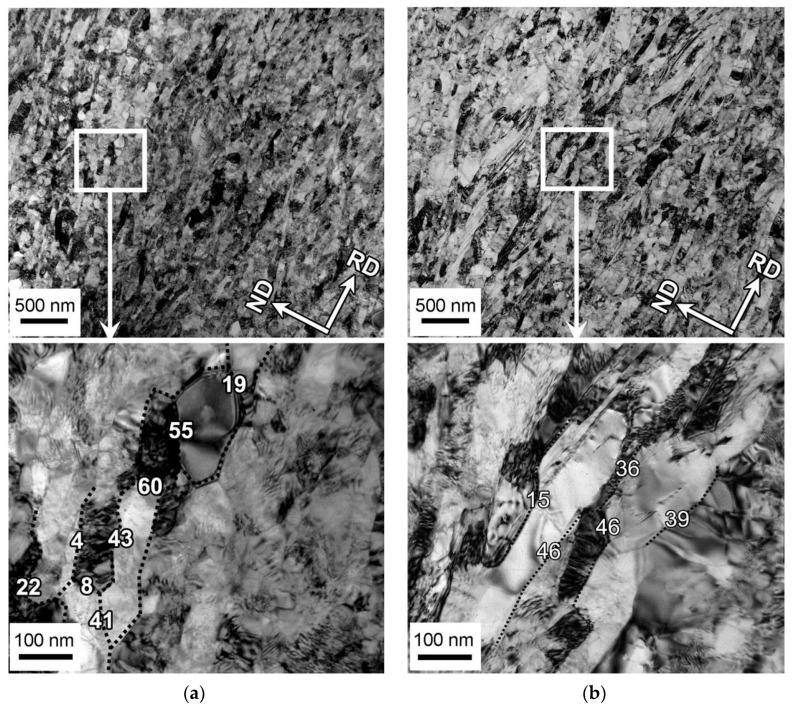
Typical fine structures evolved in a 316L-type stainless steel after cold (**a**) or warm (**b**) rolling and annealing for 30 min at 973 K.

The fine structures that developed in the cold and warm rolled samples after annealing for 30 min at 973 K are represented in Figure 3. The austenite reversal and a kind of continuous recrystallization develop concurrently and result in the formation of ultrafine-grained microstructure with varying dislocation density (Figure 3a). Similar microstructure develops during annealing of the warm rolled sample (Figure 3b). It should also be noted that the annealed microstructures evolved in the cold/warm rolled samples consist of elongated crystallites bonded by various boundaries with low-to-high angle misorientations (s. enlarged portions in Figure 3). This is a typical of continuous post-dynamic recrystallization [24].

**Figure 4:**
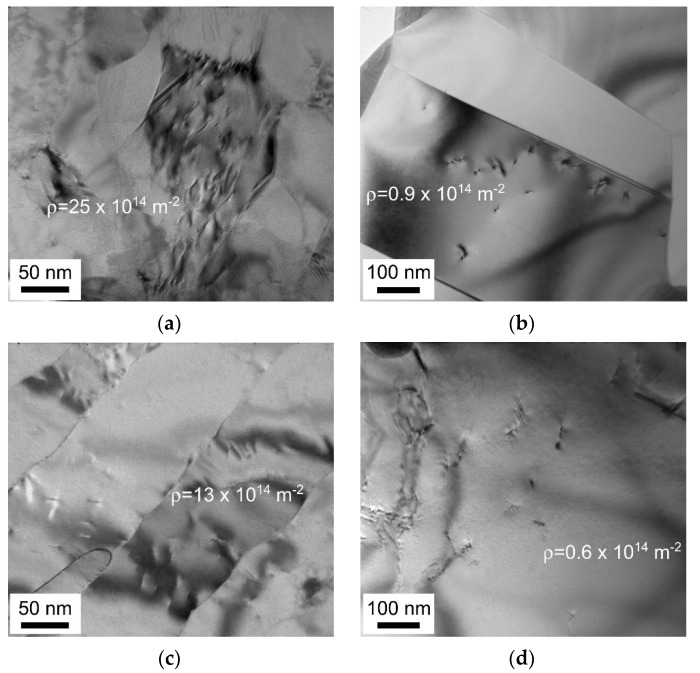
Representative transmission electron microscopy (TEM) images of dislocation substructures (with corresponding dislocation densities) that remained in a 316L-type stainless steel after cold (**a**,**b**) or warm (**c**,**d**) rolling and 30 min annealing at 973 K (**a**,**c**) or 1073 K (**b**,**d**).

Typical examples of dislocation substructures in the cold/warm rolled and annealed samples are shown in Figure 4. The dislocation densities as calculated in the presented TEM images are indicated in the corresponding figures. Both cold and warm rolled samples are characterized by a rather high dislocation density of above 10^15^ m^−2^ in average after annealing at 973 K (Figure 4a,c, Table 1). Continuous recrystallization followed by grain growth at 1073 K leads to a decrease in the dislocation density to about 10^13^ m^−2^. It is worth noting that relatively high dislocation density of 0.9 × 10^14^ m^−2^ is observed close to annealing twin as shown in Figure 4b. Despite pronounced grain coarsening upon annealing at 1073 K, therefore, the recrystallized grains are characterized by certain dislocation density, which at least an order of magnitude is higher than that in primary (conventionally) recrystallized microstructures [24]. The excess dislocations can be observed in the annealed grains as individual dislocations as well as dislocation arrays.

**Figure 5:**
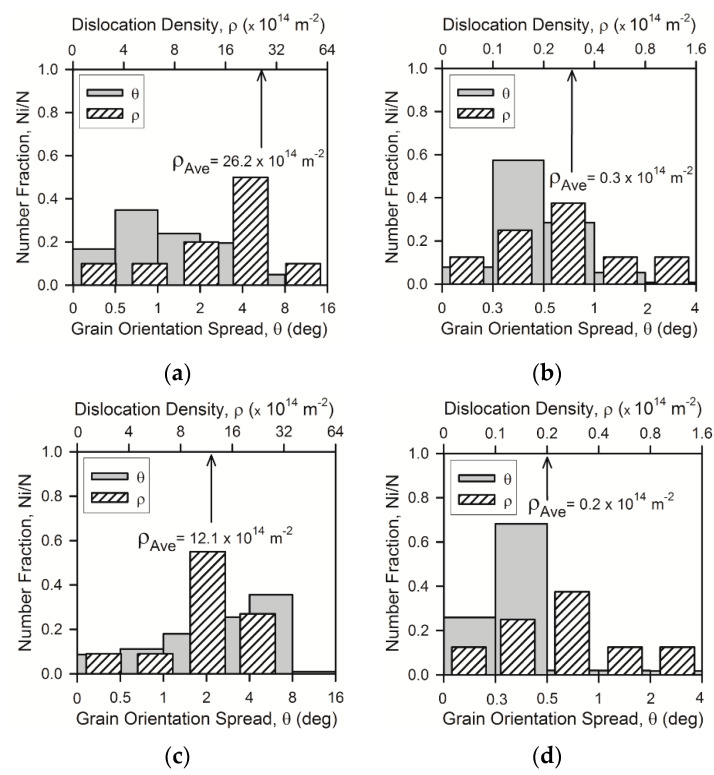
Distributions of the grain orientation spread and the dislocation density in a 316L-type steel subjected to cold rolling (**a**,**b**) or warm rolling (**c**,**d**) and subsequent annealing for 30 min at 973 K (**a**,**c**) or 1073 K (**b**,**d**).

The residual dislocation density is not the same in different annealed grains. Figure 5 shows the distribution of dislocation density in the annealed microstructures as observed by TEM along with GOS distribution revealed by OIM. The latter represents the local curvature of crystal lattice within a grain and, thus, can also be related to the dislocation substructure. Simultaneous consideration of residual dislocations/substructures on micro-scale (TEM) and meso-scale (OIM) enhances the confidence and generality of conclusions. Irrespective of processing conditions, the residual dislocation density in the all samples subjected to cold or warm rolling followed by annealing at different temperatures varies locally in a rather wide range. As should be expected, the values of local dislocation density decrease with an increase in the annealing temperature. As a result, the variation range of the residual dislocation density narrows with a progress in recrystallization because of disappearance of local high dislocation densities first. Nevertheless, the dislocation density varies locally in a wide range even in apparently well recrystallized microstructures after annealing at 1073 K. It should also be noted that in spite of variations of dislocation densities in different annealed grains, the distributions of both the dislocation density (revealed by TEM) and the grain orientation spread (obtained by OIM) in all samples are characterized by distinct maximums against rather narrow intervals that enables one to utilize the average values of residual dislocation density. Similar to the grain size, the average dislocation density is an important parameter characterizing the annealed microstructures that should be taken into account while evaluating the structure-property relationship in the present steel samples.

### 3.2. Tensile Behavior

**Figure 6:**
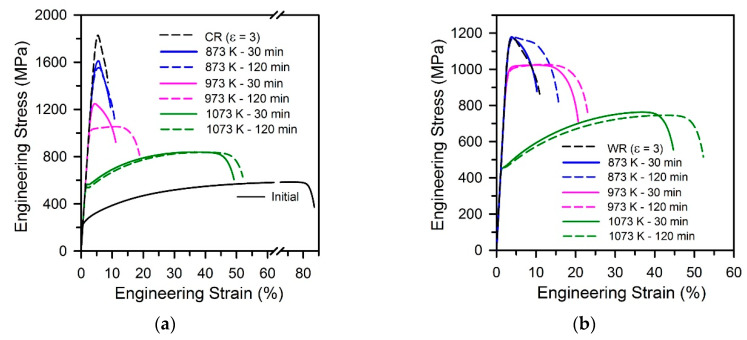
Engineering stress-strain curves for a 316L-type stainless steel processed by cold (**a**) or warm (**b**) rolling and then annealed under the indicated conditions.

The engineering stress-strain curves obtained by tensile tests of the steel samples subjected to warm and cold rolling to a strain of 3 and subsequent annealing at 873–1073 K are shown in Figure 6. Some tensile properties of the samples subjected to different annealing conditions are listed in Table 1. The tensile behavior of samples after annealing at 873 K during 30 min is characterized by a sharp stress maximum at very small strains followed by rapid necking and failure that is much similar to that after large strain cold rolling [3]. An increase in annealing time to 120 min at 873 K leads to an increase in total elongation from 8% to 14%, wherein the yield strength does not change remarkably (σ_0.2_ = 1080 MPa) in the warm rolled sample. On the other hand, the cold rolled sample is characterized by somewhat decrease in the yield strength from 1385 MPa to 1330 MPa with an increase in annealing time at 873 K. A beneficial combination of the strength and plasticity is observed after annealing for 120 min at 973 K. This treatment results in σ_0.2_ = 900 MPa, δ = 20% in the warm rolled sample and σ_0.2_ = 960 MPa, δ = 17% in the cold rolled one. These properties are associated with the ultrafine-grained annealed microstructure, in which the grains with relatively high dislocation density provide the high strength, whereas the grains with lowered dislocation density are responsible to the strain-hardening, similar to UFG dual-phase (ferrite + marteniste) steels [10]. The samples annealed at 1073 K exhibit ordinary tensile behavior, which is typical of recrystallized steels [4], although the fine grained microstructures in these samples provide rather high yield strength well above 400 MPa.

## 4. Discussion

### 4.1. Annealing Behavior

**Figure 7:**
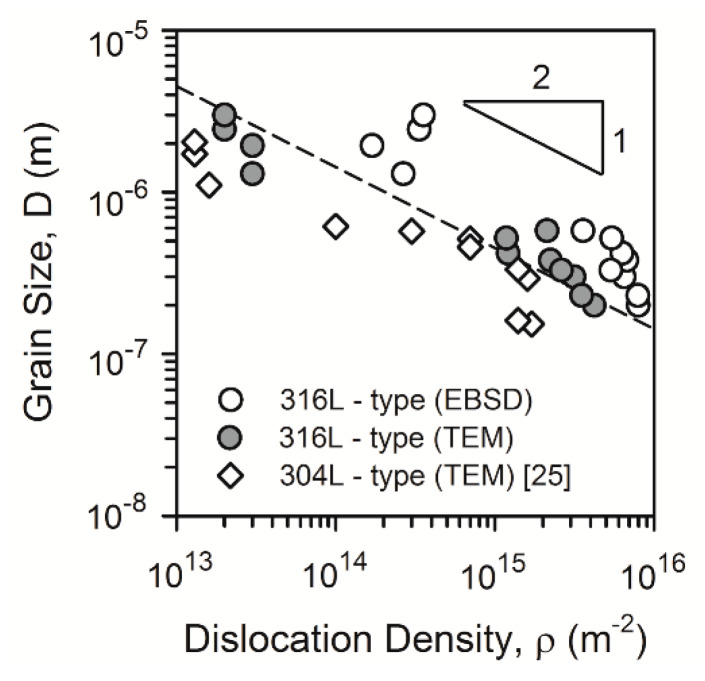
Relationships between the grain size (D) and the dislocation density (*ρ*) in a 316L-type and 304L-type stainless steels processed by cold or warm rolling and subsequent annealing.

Figure 7 represents the relationship between the annealed grain size and the dislocation density in the present samples as well as the data for a 304L-type stainless steel [25]. The dislocation density calculated as *ρ* = 2θ_KAM_/(bd), where θ_KAM_ is the kernel average misorientation (KAM) in OIM, b and d are the Burgers vector and the OIM step size, respectively, is also displayed in Figure 7. KAM gives somewhat overestimated dislocation density because of, probably, low-angle dislocation subboundaries, which were omitted in the TEM calculations. Nevertheless, this figure suggests a unique relationship between the grain size and the dislocation density for the UFG microstructures evolved by large strain cold/warm working followed by continuous recrystallization. Namely, the grain size can be expressed by a power law function of the dislocation density with an exponent of −0.5, that is,

(1) lg(D)=C−0.5lg(ρ)

or *D* = C_0_ *ρ* ^−0.5^, where C_0_ = 14 for the dashed line in Figure 7.

According to the model for normal (continuous) grain growth, originally proposed by Burke and Turnbull [26], the rate of grain boundary migration (*V*) depends on the boundary surface energy (γ):

(2) $$V=\frac{K\;\gamma \;V_{A}}{r}\;V$$

where K is a constant, V_A_ is the atomic volume and *r* is the radius of the boundary curvature. Assuming that the boundary curvature radius is proportional to the grain diameter (*r* ~ *D*) and taking d*D*/d*τ* ~ *V* (here *τ* is the annealing time), the following dependence for the annealing grain size was obtained.

(3) $$D^{2}-D_{0}^{2}=K\;\gamma \;V_{A}\;\tau$$

where *D*_0_ is the initial grain size. In the case, when the initial grain size is much smaller than annealed one, a power law relationship with a grain growth exponent of 2 can be obtained, that is, *D* ~ *τ*^0.5^.

On the other hand, in accordance with a dislocation annihilation recovery model as elaborated by Humphreys and Hatherly [27], decreasing the dislocation density can be expressed as follows:

(4) $$\frac{d\rho }{d\tau }=-C_{R}\rho^{2}$$

or otherwise *ρ*^−1^ – *ρ*_0_^−1^= C_R_ *τ*, where C_R_ is a coefficient and ρ_0_ is the initial dislocation density right after cold/warm working. Since annealed dislocation density is much lower by several orders of magnitude than that right after cold/warm working, that is, *ρ* << *ρ*_0_, *ρ* ~ τ^−1^. Then, combining the time dependencies for grain growth and dislocation recovery, the grain size can be expressed by a power law function of dislocation density with an exponent of −0.5, that is, *D* ~ *ρ*^−0.5^, which coincides with the experimental results shown in Figure 7.

### 4.2. Structural Strengthening

**Figure 8:**
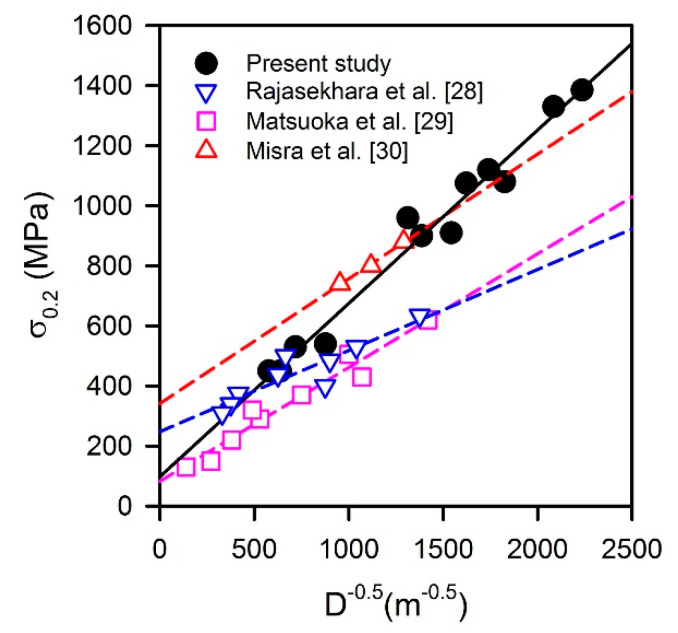
Relationships between the yield strength (σ_0.2_) and the grain size (D) in stainless steels processed by cold/warm working and annealing [28,29,30].

The relationship between the yield strength (σ_0.2_) and the grain size is represented in Figure 8. The present steel samples processed by warm or cold rolling and subsequent annealing obey the following Hall-Petch-type relationship:

(5) $$\sigma_{0.2}=100+0.58D^{-0.5}$$

The data in Figure 8 suggest that there should be an additional strength contributor for the present samples, since the grain size strengthening factor has a large value of K_y_ = 0.58 MPa m^0.5^, which is significantly large than those of 0.27–0.34 MPa m^0.5^ in other studies on austenitic stainless steels with statically recrystallized microstructures [28,29]. Relatively large strengthening factor of about 0.42 MPa m^0.5^ has only been observed in UFG steel with a grain size below 1 μm developed by a rapid reversal annealing for 1 s [30].

The additional strength contribution in the present study can be attributed to the high dislocation density. The annealed UFG samples are characterized by rather high dislocation densities remaining from the work hardened state, because austenite reversal by a shear mechanism and continuous static recrystallization do not remove the dislocations completely as compared to primary discontinuous recrystallization [24]. The high dislocation densities contribute to the strengthening and are responsible for a deviation of Hall-Petch-type relationship from that observed for conventionally recrystallized steels with relatively coarse grains. Assuming the strength contributions from grain boundaries and dislocations being independent and linearly additive, the modified relationship for the offset yield strength should include an additional term for the dislocation strengthening, which is much similar to Taylor-type equation [31].

(6) $$\sigma_{0.2}-\sigma_{0}=K_{y}D^{-0.5}+\alpha \;G\;b\;\rho^{0.5}\sigma$$

Here *σ*_0_ is the strength of dislocation-free single crystal (180 MPa), G is the shear modulus (81000 MPa), b is the Burgers vector (0.26·nm) and α is a numerical factor [7].

**Figure 9:**
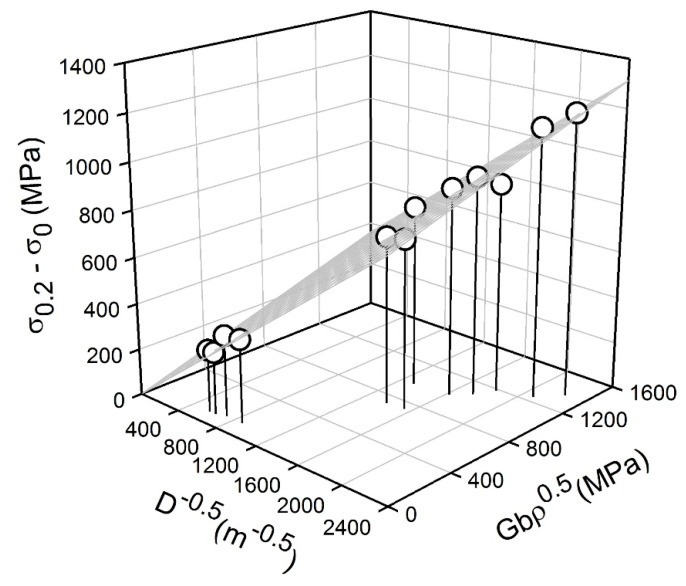
Relationship between the strength increment (*σ*_0.2_−*σ*_0_), the grain size (*D*) and dislocation density (*ρ*) strengthening in a 316L-type stainless steel processed by cold or warm rolling and subsequent annealing.

Figure 9 shows the results of strength calculation through Equation (6). The best fit by the plane in Figure 9 is obtained with K_y_ = 0.4 MPa m^0.5^ and α = 0.22. The former is quite close to those reported in other studies on grain size strengthening [28,29,30], whereas the latter is remarkably smaller than those of 0.5 to 1.5, which have been frequently used to evaluate the dislocation strengthening [32,33]. This difference in the dislocation strengthening factor can be attributed to the dislocation rearrangement in the present steel samples owing to recovery and continuous recrystallization during annealing that could significantly weaken the dislocation strengthening as compared to work hardened materials, where disclination-type irregular dislocation arrays create long-range internal stress fields [34]. It should be noted in Figure 9 that an almost linear relationship between the grain size strengthening and the dislocation strengthening, that is, *D*^−0.5^ ~ Gb*ρ*^0.5^, complicates the calculation of meaningful K_y_ and α. The following relationship, α = 0.88–1.6 K_y_, can be derived from Figure 9 in the present study. Hence, an increase in K_y_ is compensated by a decrease in α and vice versa. Then, the strength can be calculated by using K_y_ and α varying in a wide range, keeping in mind that an increase in one coefficient decreases another. Probably, this phenomenon was met in other studies elaborating the grain size and dislocation density strengthening. Table 2 presents K_y_ and α reported for the strength calculation through Equation (6) for various stainless steels and similar alloys [7,19,23,35,36,37,38,39,40,41]. Those factors were obtained by either approximation of experimental data or just taken from literature data. With rare exception, the pairs of K_y_ and α are characterized by quite different values, that is, if one of coefficients is large, another is small. Large α was frequently used owing to well-known theoretical consideration [42]. As a consequence, K_y_ should be relatively small to compensate for a possible overestimation of dislocation strengthening.

**Figure 10:**
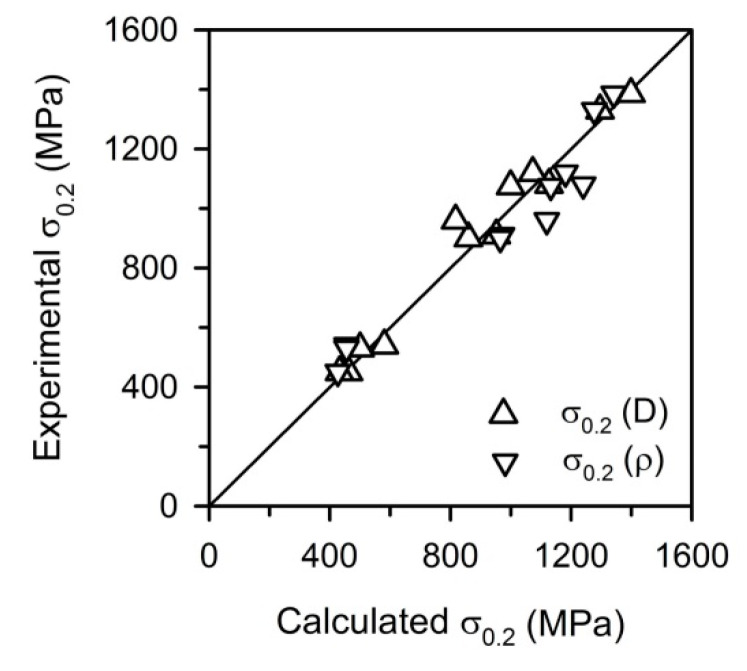
Relationship between the calculated and experimental yield strength (σ_0.2_) in a 316L-type stainless steel processed by cold or warm rolling and subsequent annealing.

The obtained relationship between the grain size and the dislocation density (Figure 7) suggests that the strength of UFG metallic materials processed by large strain warm/cold working and subsequent annealing can be evaluated by using either grain size or dislocation density, taking *D*^−0.5^ = C_0_^−0.5^*ρ*^0.25^ or *ρ*^0.5^ = C_0_/*D*. The relationship between the experimental yield strengths and those calculated by Equation (6) taking either the grain size or dislocation density as a structural parameter in accordance with obtained relationship from Figure 7 is shown in Figure 10. It is clearly seen that calculated yield strengths match well the experimental ones that validates the speculation above. The obtained relationship expands our understanding of the mechanisms of post-dynamic continuous recrystallization in metallic materials and can be used for strength prediction of UFG steels and alloys processed by large strain deformation followed by annealing. The grain size seems to be the most suitable parameter for the strength calculation because it is easier accessible one as compared to the dislocation density.

## 5. Conclusions

Ultrafine-grained (UFG) microstructures and their effect on the mechanical properties of a 316L-type stainless steel subjected to cold or warm rolling at 293 K or 573 K, respectively, followed by annealing at 873–1073 K were studied. The main results can be summarized as follows.

- The austenite reversal (in the cold rolled samples) and continuous recrystallization followed by grain growth led to the UFG microstructures with a grain size of about 0.5 μm and a dislocation density of about 10^15^ m^−2^ after annealing at 973 K of the cold or warm rolled 316L-type steel. These UFG microstructures led to a combination of high yield strength (σ_0.2_ > 900 MPa) and satisfactory plasticity (δ > 15%).
- A power law function was obtained between the grain size (D) and the dislocation density (*ρ*) in the form of D ~ *ρ*^−0.5^ for the UFG 316L-type steel processed by cold or warm rolling and subsequent annealing. The observed relationship meets the model conditions for normal grain growth along with dislocation annihilation recovery.
- The yield strength could be expressed by a modified Hall-Petch-type relationship including a term for dislocation strengthening, assuming that the strength contributions from grain boundaries and dislocations are independent and linearly additive. Taking the mutual relationship between the grain size and the dislocation density, the yield strength could be accurately evaluated by using a single arbitrary selected structural parameter.

## Figures and Tables

**Table 1:** The grain size (D), the dislocation density (*ρ*), the yield strength (σ_0.2_), the ultimate tensile strength (UTS) and the total elongation (δ) of a 316L-type stainless steel subjected to cold rolling (CR) or warm rolling (WR) and subsequent annealing at indicated conditions.

Condition	D, μm	*ρ*, 10^15^ m^−2^	σ_0.2_, MPa	UTS, MPa	δ, %
Initial	21 ± 4	0.002 ± 0.001	235 ± 20	585 ± 20	86 ± 3
CR + 873K (30 min)	0.20 ± 0.02	4.21 ± 0.50	1385 ± 10	1630 ± 10	6 ± 2
CR + 873K (120 min)	0.23 ± 0.02	3.51 ± 0.40	1330 ± 10	1550 ± 10	8 ± 2
CR + 973K (30 min)	0.33 ± 0.03	2.62 ± 0.30	1120 ± 10	1250 ± 10	9 ± 2
CR + 973K (120 min)	0.58 ± 0.06	2.13 ± 0.30	960 ± 10	1055 ± 10	17 ± 2
CR + 1073K (30 min)	1.30 ± 0.15	0.03 ± 0.01	540 ± 10	840 ± 10	48 ± 3
CR + 1073K (120 min)	1.94 ± 0.20	0.03 ± 0.01	530 ± 10	835 ± 10	50 ± 3
WR + 873K (30 min)	0.30 ± 0.03	3.15 ± 0.50	1080 ± 10	1175 ± 10	8 ± 2
WR + 873K (120 min)	0.38 ± 0.04	2.23 ± 0.40	1075 ± 10	1175 ± 10	14 ± 2
WR + 973K (30 min)	0.42 ± 0.04	1.21 ± 0.30	910 ± 10	1025 ± 10	19 ± 2
WR + 973K (120 min)	0.52 ± 0.05	1.18 ± 0.30	900 ± 10	1025 ± 10	21 ± 2
WR + 1073K (30 min)	2.45 ± 0.25	0.02 ± 0.01	450 ± 10	760 ± 10	43 ± 3
WR + 1073K (120 min)	3.00 ± 0.30	0.02 ± 0.01	450 ± 10	745 ± 10	51 ± 3

**Table 2:** The strengthening factors, Ky and α, for ultrafine-grained (UFG) austenitic steels and some fcc-alloys subjected to large strain deformation.

Material	Ky (MPa m^0.5^)	α	Reference
304L austenitic stainless steel after cold rolling	0.24 (experiment)	0.73 (experiment)	[7]
Nickel after cold rolling	0.158 (literature)	0.72 (literature)	[19]
316L Austenitic stainless steel after cold rolling	0.047 (experiment)	0.64 (experiment)	[23]
Aluminum after accumulative roll-bonding and annealing	0.041 (experiment)	0.72 (literature)	[35]
S304H austenitic stainless steel after warm-to-hot rolling	0.12 (experiment)	0.7 (experiment)	[36]
S304H austenitic stainless steel after cold rolling	0.2 (experiment)	0.77 (experiment)	[37]
321 austenitic stainless steel after equal channel angle pressing and rolling	0.3 (experiment)	0.9 (literature)	[38]
Copper alloy after large strain warm deformation	0.05 (experiment)	1.0 (experiment)	[39]
304 austenitic stainless steel after laser welding	0.317 (literature)	0.6 (literature)	[40]
Aluminum alloy after cold rolling and annealing	0.045 (experiment)	0.72 (literature)	[41]

## List of Abbreviations and Symbols

CR	Cold rolling
CSL	Coincident site lattice
EBSD	Electron back scattering diffraction
GOS	Grain orientation spread
KAM	Kernel average misorientation
ND	Normal direction
OIM	Orientation imaging microscopy
TD	Transverse direction
TEM	Transmission electron microscope
UFG	Ultrafine-grain
UTS	Ultimate tensile strength
WR	Warm rolling
b	Burgers vector
D	Grain size
d	OIM step size
Ky	Grain size strengthening factor
r	Radius of boundary curvature
G	Shear modulus
V	Rate of grain boundary migration
V_A_	Atomic volume
α	Numerical factor
γ	Boundary surface energy
δ	Total elongation
*ρ*	Dislocation density
σ_0.2_	Yield strength
*σ*_0_	Strength of dislocation-free single crystal
τ	Annealing time
